# New records of the critically endangered fern *Grammitisazorica* (Polypodiaceae)

**DOI:** 10.3897/BDJ.10.e77948

**Published:** 2022-01-28

**Authors:** Rui Bento Elias, Fernando Pereira

**Affiliations:** 1 CE3C – Centre for Ecology, Evolution and Environmental Changes/Azorean Biodiversity Group and Universidade dos Açores - Faculdade de Ciências Agrárias e do Ambiente, 9700-042, Angra do Heroísmo, Açores, Portugal, Angra do Heroísmo, Portugal CE3C – Centre for Ecology, Evolution and Environmental Changes/Azorean Biodiversity Group and Universidade dos Açores - Faculdade de Ciências Agrárias e do Ambiente, 9700-042, Angra do Heroísmo, Açores, Portugal Angra do Heroísmo Portugal

## Abstract

*Grammitisazorica* (H. Schaef.) H. Schaef. is a critically endangered epiphytic fern, endemic to the Azores Islands. Until 2013, its presence was only confirmed on Flores Island. Our new records expand the distribution of this species from one to three islands and the altitudinal range to 640 – 1130 m a.s.l. Nevertheless, the fact that only four additional individuals were found confirms the rarity of this fern. Our new data also highlight the importance of montane forests and woodlands as hotspots of biodiversity in the Azores and the need to increase the protection status of all areas with remnant natural forest patches.

## Introduction

**Figure 1.**
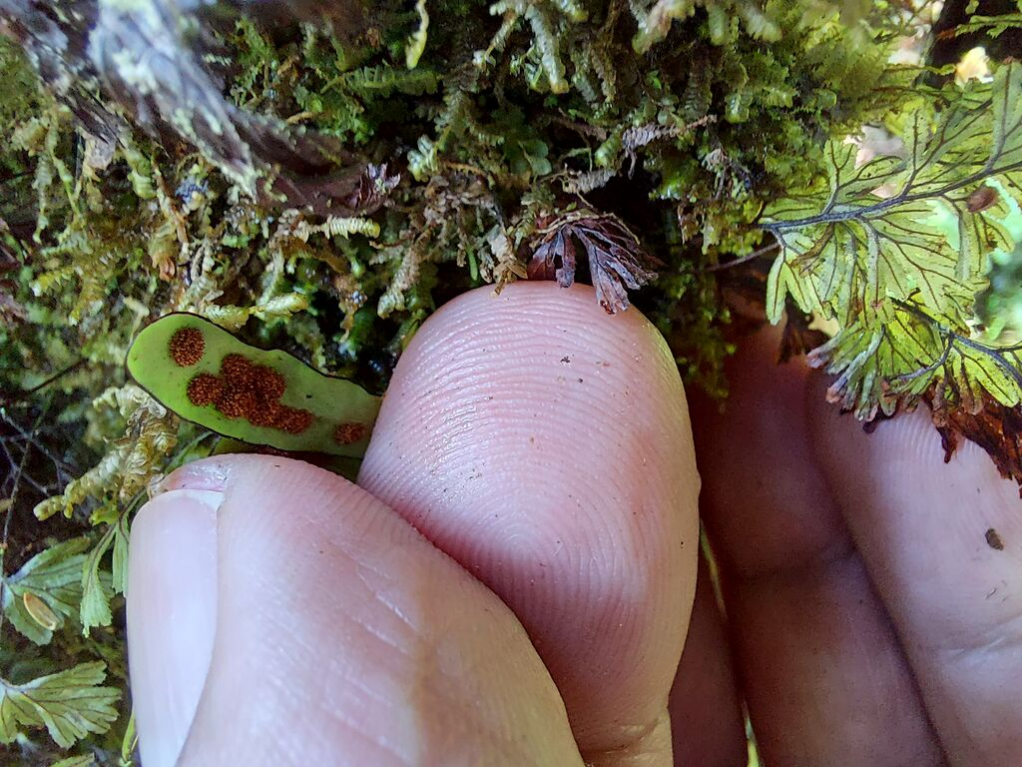
Fertile frond of *Grammitisazorica* with large sori (Photo by Rui Elias).

*Grammitis* Sw. is a genus of ferns from the family Polypodiaceae (PPG 2016) with around 30 species occurring mostly in the Tropics (Hassler 2021). *Grammitisazorica* is a notable exception since it is endemic to the temperate islands of the Azores (Schaefer 2005). The basionym of this species, Grammitismarginella(Sw.)Sw.subsp.azorica H.Schaef., was first described in 2001 (Schaefer 2001) from specimens observed in Flores. This is a very small epiphytic fern, mainly of old trees of *Juniperusbrevifolia* (Seub.) Antoine. It has simple fertile fronds, up to 60 mm, with entire, black margins and large, globose sori (Fig. 1). Until 2013, the only confirmed records of this species were 22 individuals from Flores Island (Schaefer 2001). Nevertheless, in 1987, Hansen (Hansen 1992) saw, in Pico Island, a small fern, similar to *Ceradeniajungermannioides* (Klotzsch) L.E.Bishop, but with black margins, which he provisionally identified as *Grammitisebenina* (Maxon) Tardieu. Given its rarity, the IUCN classified *Grammitisazorica* as critically endangered (García Criado et al. 2017).

## New records

**Figure 2.**
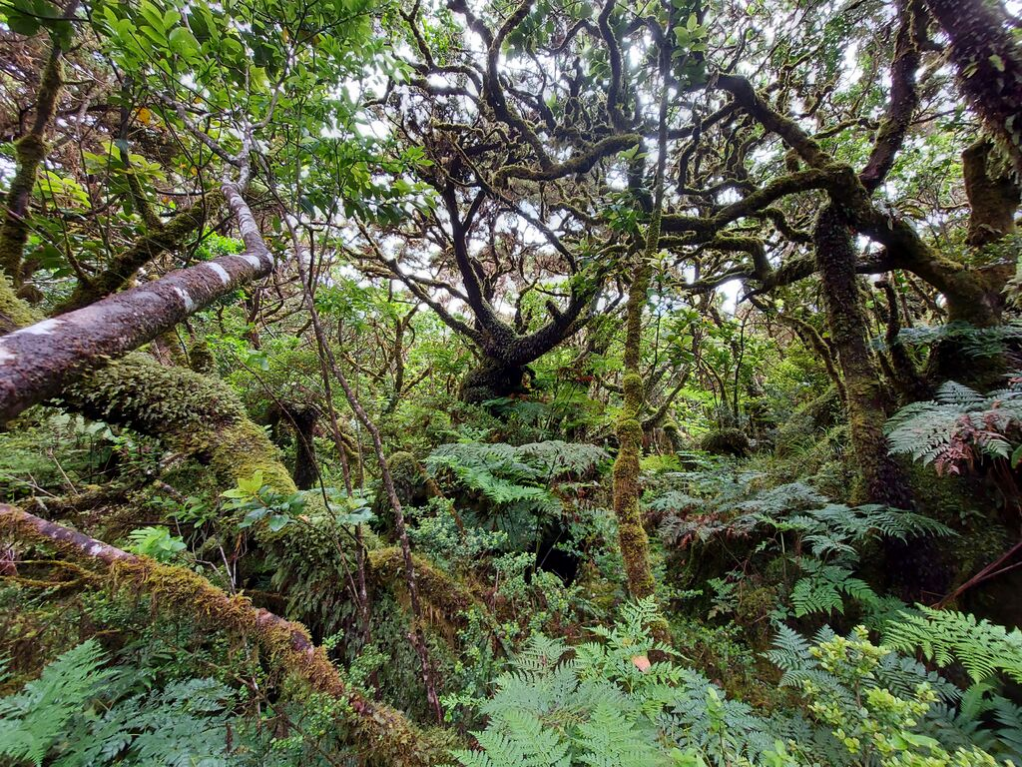
*Juniperus-Ilex* montane forest in Terra Brava (Terceira Island) (Photo by Rui Elias).

In 2013, we recorded two specimens for the first time in Terceira Island (Table 1). These records were never published, but were added to the azoresbioportal database (Borges et al. 2018). The specimens were 3 km apart, in two distinct Natural Reserves (Biscoito da Ferraria e Pico Alto and Terra Brava e Criação das Lagoas). These Natural Reserves harbour some of the most pristine *Juniperus-Ilex* montane forests of the Azores (Elias et al. 2016) (Fig. 2). Both specimens were on small old *Juniperusbrevifolia* trees.

**Figure 3.**
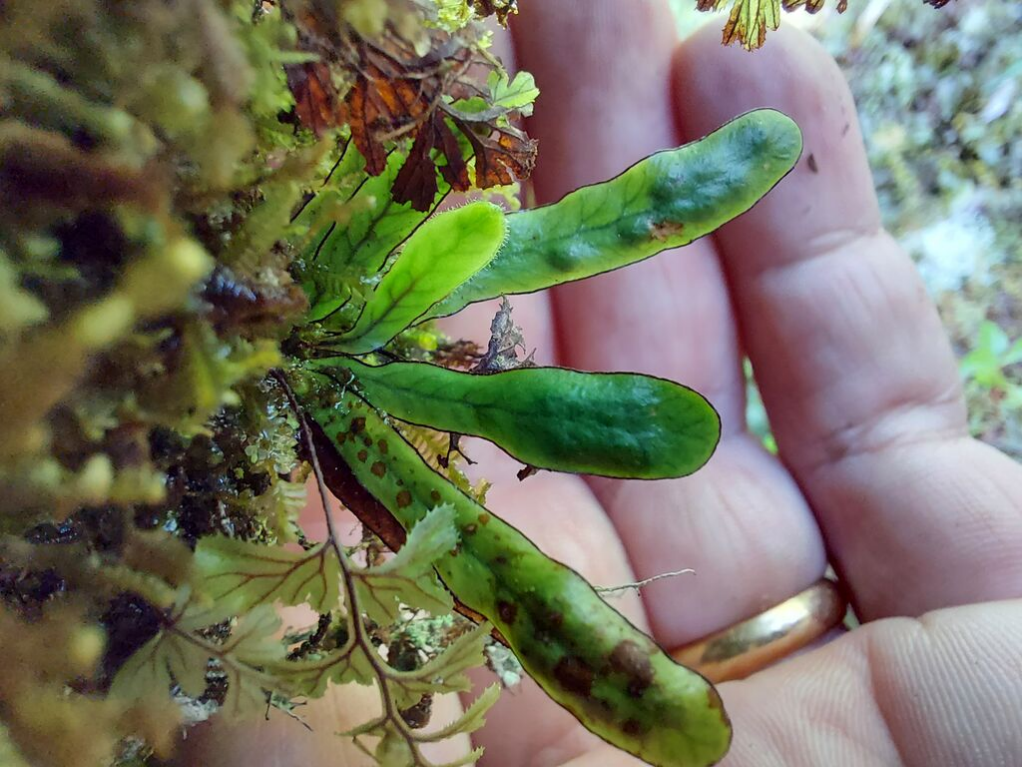
*Grammitisazorica* in Caveiro (Pico Island) (Photo by Rui Elias).

Very recently, in July 2021, during an expedition, we found two additional plants in Pico Island (Fig. 3). The first was in a protected landscape area neighbouring the Pico Mountain Natural Reserve. The second was 19 km to the east, in Caveiro Natural Reserve. Both were also in *Juniperus-Ilex* montane forests, on *Juniperusbrevifolia* trunks.

## Concluding remarks

Our new data highlight the importance of montane forests and woodlands as hotspots of biodiversity in the Azores. In fact, the few known individuals of *Grammitisazorica* occur in *Juniperus* montane woodlands, in Flores and *Juniperus-Ilex* montane forests, in Terceira and Pico. Our new records expand the distribution of this species from one to three islands and the altitudinal range from 650 – 800 to 640 – 1130 m a.s.l. Nevertheless, given the distribution of montane forests and woodlands, its core altitudinal range should be 600 – 1000 m a.s.l. In fact, the individual found at the highest altitude (Lomba do Fogo) is in a sheltered forest, located inside a small cinder cone from a historical eruption.

The fact that only four new individuals were found, in spite of our continuous efforts for the past nine years, confirms the rarity of this species and the need to preserve the remaining montane forests of the Azores. In this aspect, it is of the utmost importance to increase the protection status of Lomba do Fogo in Pico. This site has a series of cinder cones and crevices with stunning natural forests, harbouring several endemic species, many of them rare or very rare. Lomba do Fogo is inside Pico Natural Park, but is under one of the lowest protection levels (Protected landscape). As it is at the base of Pico Mountain, this site could easily be integrated into the Pico Mountain Natural Reserve, thus gaining a much more adequate protection status.

As many montane forests and woodlands, especially in Flores, Terceira and Pico, are very difficult to access, there are still many unexplored areas. We must maintain our efforts to find new individuals and increase our knowledge about the ecology of this species. Our new data also highlight the need to increase the protection status of all areas with remnant natural forest patches, since this is the only way to ensure the survival of many endangered species.

## Figures and Tables

**Table 1.** New *Grammitisazorica* records for Terceira and Pico Islands. To protect them from collectors, we do not indicate the precise coordinates of the individuals, but only the coordinates of the respective 100 x 100 m UTM grid centres.

Island	Site	Date	Coordinates	Altitude (m a.s.l.)
Terceira	Terra Brava	11/07/2013	38.735606°N, -27.200750°W	640
	Morro Assombrado	15/07/2013	38.760822°N, -27.220392°W	670
Pico	Lomba do Fogo	13/07/2021	38.484837°N, -28.410808°W	1130
	Caveiro	17/07/2021	38.436690°N, -28.201374°W	910
